# Motor evoked potentials for multiple sclerosis, a multiyear follow-up dataset

**DOI:** 10.1038/s41597-022-01335-0

**Published:** 2022-05-16

**Authors:** Jan Yperman, Veronica Popescu, Bart Van Wijmeersch, Thijs Becker, Liesbet M. Peeters

**Affiliations:** 1 Biomedical Research Institute (BIOMED), Hasselt University, 3500 Hasselt, Belgium; 2 Data Science Institute (DSI), Hasselt University, 3500 Hasselt, Belgium; 3 University MS Center (UMSC), Hasselt University, 3500 Hasselt, Belgium; 4 Theoretical Physics, Hasselt University, 3500 Hasselt, Belgium; 5 University MS Center (UMSC), Noorderhart Hospital, 3900 Pelt, Belgium; 6 I-Biostat, Data Science Institute (DSI), Hasselt University, 3500 Hasselt, Belgium

**Keywords:** Predictive markers, Multiple sclerosis

## Abstract

Multiple sclerosis (MS) is a chronic disease affecting millions of people worldwide. Through the demyelinating and axonal pathology of MS, the signal conduction in the central nervous system is affected. Evoked potential measurements allow clinicians to monitor this process and can be used for decision support. We share a dataset that contains motor evoked potential (MEP) measurements, in which the brain is stimulated and the resulting signal is measured in the hands and feet. This results in time series of 100 milliseconds long. Typically, both hands and feet are measured in one hospital visit. The dataset contains 5586 visits of 963 patients, performed in day-to-day clinical care over a period of 6 years. The dataset consists of approximately 100,000 MEP. Clinical metadata such as the expanded disability status scale, sex, and age is also available. This dataset can be used to explore the role of evoked potentials in MS research and patient care. It may also be used as a benchmark for time series analysis and predictive modelling.

## Abstract (summary)

**Table:** 

Measurement(s)	Abnormal upper-limb motor evoked potentials • Abnormal lower-limb motor evoked potentials • Kurtzke Expanded Disability Status Scale Clinical Classification
Technology Type(s)	Transcranial Magnetic Stimulation • performing a clinical assessment
Factor Type(s)	Date of birth • Sex • Measurement date
Sample Characteristic - Organism	Homo sapiens
Sample Characteristic - Location	Belgium

## Background & Summary

Multiple sclerosis (MS) is an incurable, chronic disease characterized by the disruption of electrical signal conduction over axons in the central nervous system. One of the main reasons for this disruption is the loss of the myelin sheath^1^. This leads to a myriad of symptoms including balance impairment, optical neuritis, fatigue, muscle weakness, cognitive symptoms, and more.

There has been significant progress in the development of disease-modifying treatments that can suppress disease worsening^2^. The main method of quantifying disability in MS is the Kurtzke Expanded Disability Status Scale (EDSS)^3^. Many other measures exist, with mainly the Multiple Sclerosis Functional Composite (MSFC) seeing an increase in use alongside EDSS^4^. EDSS is a score that runs from 0 (normal neurological exam) to 10 (death due to MS), in increments of 0.5.

It has a number of weaknesses such as low reliability and sensitivity to change, see^4^ for a review of the psychometric properties of EDSS. It is nonetheless the most often used and therefore the most consistently available measure in retrospective data.

**Fig. 1:**
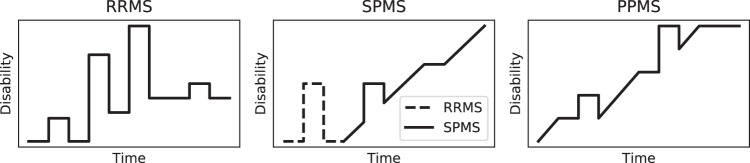
Illustration of typical disease courses for the various phenotypes of MS. The vertical axis shows the disability, which is often quantified by the EDSS score. Abbreviations used: Relapsing-remitting MS (RRMS), Secondary Progressive MS (SPMS), and Primary Progressive MS (PPMS).

There are 4 main MS phenotypes in use as described by^5,6^: Clinically isolated syndrome (CIS), Relapsing-remitting MS (RRMS), Secondary Progressive MS (SPMS) and Primary Progressive MS (PPMS). Typical trajectories of disability as a function of time are illustrated in Fig. 1 for all phenotypes excluding CIS.

CIS is the first clinical presentation of a disease that shows characteristics of inflammatory demyelination that could be MS, but has yet to fulfill criteria of dissemination in time^7^. These are patients that have had an episode displaying symptoms of MS, but they may or may not go on to develop MS. It is usually only after a second episode that MS is diagnosed.

RRMS is the most common disease course of MS, with about 85% of people with MS being diagnosed with this phenotype. It is characterized by recurring episodes of new or increasing neurological symptoms, occurring erratically but rarely exceeding 1.5 episodes per year^8^. In between these episodes there are periods of complete or partial recovery (remissions). Around 15% to 30% of people diagnosed with RRMS will eventually transition to SPMS^9^, which is characterized by progressive deterioration of neurological function over time.

Finally, for about 15% of people with MS, the disease course will be progressive from the onset (PPMS). In this new classification^6^, other clinical subtypes of MS have been omitted (like Progressive-Relapsing MS, Opticospinal MS, …) and were replaced by active or non-active forms of Relapsing or Progressive disease, in which active stands for clinical (relapse or progression) or radiological activity (new or enlarging lesion, contrast-enhancing lesion). This is not a pathological classification which is beyond the scope of this study. The use of electrophysiology might help to try and classify correctly, or maybe be able to aid in determining the transition from relapsing to progression as early as possible, since this has therapeutic consequences, and therefore might be subject of further study.

The conduction through the nervous system can be assessed with evoked potential (EP) measurements^10^. These types of measurements provide insight in neural conduction by stimulating the nervous system in one place, and measuring the resulting signal at some other point^11^. They can be used to predict MS related outcomes such as walking speed, cognitive impairment or fatigue^12,13^. The different EP modalities correspond to different sites of stimulus and measurement. For visual EP (VEP) the visual system is excited and conductivity is measured in the optic nerve; for somatosensory EP (SEP) the somatosensory system (touch) is excited and conductivity is measured in the brain; and for brainstem auditory EP (BAEP) the auditory system (ears) is excited and conductivity is measured at the auditory cortex. If several types of EP are available for the same patient this is referred to as a multimodal EP (mmEP). In the case of motor evoked potentials (MEP), which is the modality this dataset contains, the motor cortex (M1) is stimulated using transcranial magnetic stimulation (TMS) and the resulting signal is measured in the hands or feet.

**Fig. 2:**
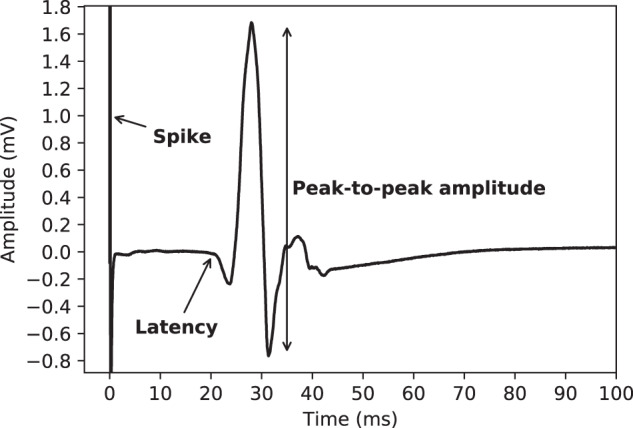
An example of a single MEP measurement. The measurement starts when the stimulation occurs in the brain and has a total duration of 100 milliseconds. The spike at the start of the measurement is a direct consequence of the stimulation. The latency is the time it takes for the signal to reach the muscle under consideration. The latency is typically higher when the motor tracts become damaged. The peak-to-peak amplitude measures the strength of the signal, which may be lower in case of damage to the motor tracts, which is typical for MS.

An example of a single MEP measurement is shown in Fig. 2. These types of measurements are usually reduced to a small set of summary variables, the main ones being the latency, the peak-to-peak amplitude, and a binary score of morphological abnormality^14,15^.

The current dataset contains MEPs obtained during about 6 years of follow-up in the Rehabilitation and MS center at Pelt, Belgium. For most of the patients the EDSS is also available.

To the best of our knowledge, there are currently no publicly available datasets that contain longitudinal follow-up of MS patients. In addition, it contains the full measurement time series, and not just the commonly used summary values such as latency and peak-to-peak amplitude. This dataset has been used to define a continuous score of morphological abnormality that agrees with the binary scoring of neurologists^16^. It has also been used to investigate whether the full MEP time series contains extra information to predict disability progression after 2 years, besides the usual summary values^17^.

Possible use cases for this dataset include time series analysis, predicting disease progression, and calculating survival curves for time to progression.

## Methods

Motor evoked potentials were recorded from the abductor pollicis brevis (APB) and abductor hallucis (AH) muscles bilaterally. Magnetic stimuli were delivered to the hand and leg areas of the motor cortex with a Magstim 200^2^ device (The Magstim Company Ltd., Whitland, UK) via a round coil with an inner diameter of 9 cm with maximal output of the stimulator of 2.2 Tesla. The signal is recorded for 100 milliseconds, starting from the moment of stimulation in the brain. The dataset contains measurements from two separate machines (cfr. Section Data Records, the *machine* field). These machines have the same hardware (the Magstim coil described above), but do differ in some of the measurement settings: The acquisition rate for machine A is 20 kHz, for B this is 19.2 kHz. Signals from machine A are filtered between 0.6 Hz and 10 kHz, while machine B has a high-pass filter with cutoff frequency 100 Hz. The measurements were performed by either the neurologists themselves, or by the specialized nurses. Which measurements were performed by whom is recorded in the *team* field (crf. Section Data Records), indicated as team A and team B for the neurologists and the nurses respectively. The measurements are not averaged across multiple trials.

The measurements are performed in a standardized way to minimize variations due to factors such as coil orientation, stimulus intensity etc.

**Fig. 3:**
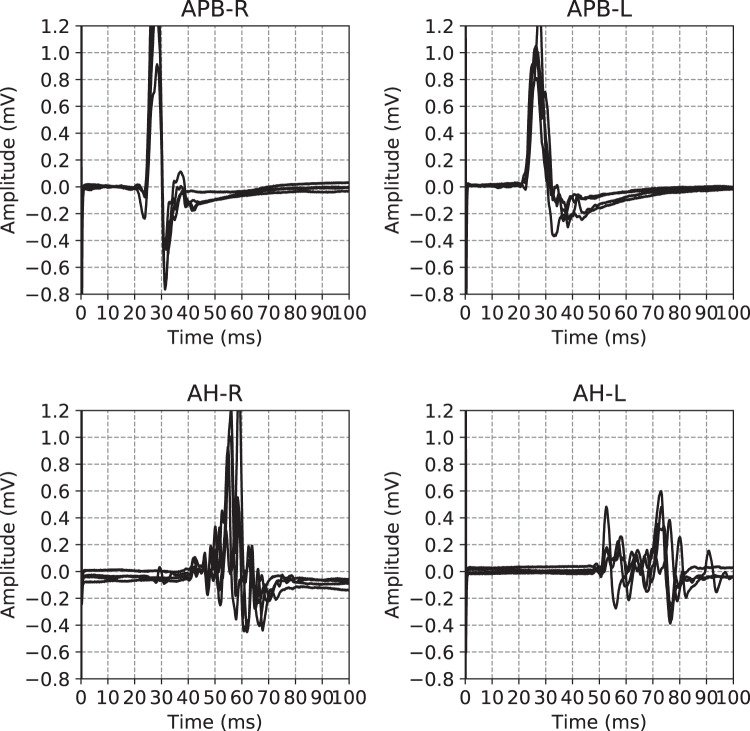
Example of the motor evoked potential time series recorded at a single hospital visit. The labels on the plot indicate the limb and the side on which the measurement was performed, Muscle Abductor Pollicis Brevis (APB) for the hands, Muscle Abductor Hallucis (AH) for the feet. The sides are indicated using R and L for right and left respectively. The time series for the same limb are the result of different magnetic excitation strengths. Adapted from Yperman *et al*.^17^.

For the hands, electrodes are placed at three places: on top of the hand at metacarpal I/II (ground), the APB muscle belly in the middle, and APB tendon at the proximal phalanx of the thumb. For the feet, electrodes are placed at three places: dorsally on top of metatarsal I (ground), the AH muscle belly in the middle and AH tendon at the proximal phalanx of the big toe. First the determination of the motor hotspot on the scalp and resting motor threshold (RMT) is done. The first excitation is at 45% (hands) or 50% (feet) of the maximal stimulator output. New stimuli are presented with an increase or decrease of 5 percentage points. The RMT is the stimulus-intensity at which 50% of the time a motor response is seen. The hotspot is the area where the largest MEP is seen at RMT. Secondly, for determination of latency and maximal amplitude, the stimulator output is increased with 5 percentage points at a time and MEPs are recorded until the amplitude stops increasing for stronger stimuli or stimulus intensity is at 70% above RMT (170% RMT) or at maximum output of the stimulator (100%). If the signal is of bad quality, as judged by the nurse, it is discarded and not recorded. An example of all the EPTS of the MEP for a single visit is shown in Fig. 3. For each limb, each excitation strength results in one EPTS.

This study was approved by the ethical commission of the University of Hasselt (CME2017/729). No consent to participate/publish was necessary since this study uses retrospective data only.

## Data Records

In this section we describe the available data in more detail. All the raw data presented in this work is available on the G-node repository associated with this work^18^.

### Cohort description

**Fig. 4:**
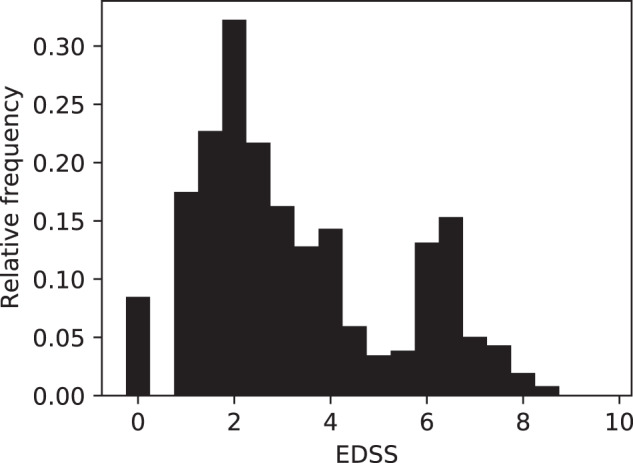
The distribution of all the EDSS (Expanded Disability Status Scale) measurements in the dataset.

The total number of patients in the dataset is 963, of whom 582 have at least one EDSS measurement. For the patients that do have EDSS measurements, and for whom the MS type was entered (263 patients), we have the following distribution of MS type: RRMS (76.0%), SPMS (19.4%), PPMS (3.4%), CIS (1.1%). The average age at the time of a visit is 49 ± 14 years. The average of the average EDSS score per patient is 3.5 ± 2. Its distribution is shown in Fig. 4.

Of the patients with more than one visit (662 patients), the average followup time is 3.4 years of MEP measurements (with on average 7 visits), and 7.4 years of EDSS measurements (with on average 13 visits). The data is collected from a clinic where many of the patients are under active treatment.

### Tables overview

**Fig. 5:**
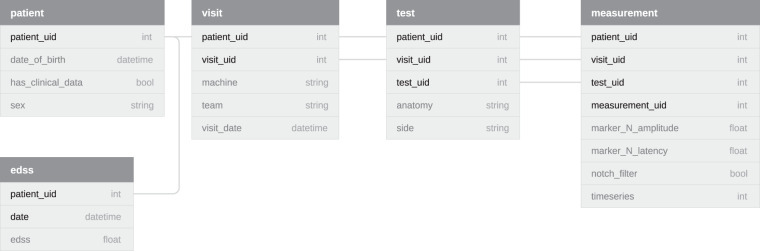
A schematic overview of the tables in the dataset. Per table, all the bold fields combine to make a unique identifier for a record in that table (composite key). Section Data Records contains descriptions of each of the fields.

In this section we provide some general numbers of the dataset. The names of the tables are self-explanatory. The relations between patients, visits, tests, and measurements are one-to-many, in that order. That is, each patient can have multiple visits. Each visit can have multiple tests. Each test can have multiple measurements. Finally, each patient can have multiple EDSS measurements. A schematic overview of the relations between the various tables is shown in Fig. 5.

To illustrate the relations between the visit, test, and measurement tables we show an example of the entries for a single visit in Fig. 3. The complete figure contains all the measurements of a particular visit. Each of the subfigures corresponds to a specific test, where a test is defined a being a set of measurements on a specific muscle, e.g. the left arm. Each of the different lines in these subplots represents an individual measurement.

The dataset contains the following tables:

- patient (963 records)
- visit (5586 records)
- test (20844 records)
- measurement (96290 records)
- edss (7414 records)

### Field descriptions

#### Table: patient

This table contains some metadata of the patients on whom the measurements were performed. Not all patients have EDSS measurements on record, usually because they only came in for a second opinion. This table is contained in the file patient.csv.

**Key fields:**

- **patient_uid**: A unique identifier for a patient. Range: 0–964.

**Other fields:**

- **date_of_birth**: The date of birth of the patient, accurate to 5 years. Format: YYYY-MM-DD. Missing values for this field: 3.84%. Average age at time of visit: 49 ± 14 years.
- **has_edss_measurements**: Whether there is are EDSS-measurements available for this patient. Missing values for this field: 0.00%. Possible values: True (60.44%), False (39.56%).
- **sex**: The sex of the patient. Missing values for this field: 4.36%. Possible values: Female (70.68%), Male (29.32%).

#### Table: visit

This table contains the metadata of the hospital visits, specifically visits where one or more MEPs were recorded. This table is contained in the file visit.csv.

**Key fields**:

- **patient_uid** - cfr. patient_uid in Table patient.
- **visit_uid**: A unique identifier for a visit. It is only unique in conjunction with the patient uid. Range: 0–25.

**Other fields**:

- **machine**: Indicates which of the two machines the measurement was performed on. Missing values for this field: 0.00%. Possible values: B (74.81%), A (25.19%).
- **team**: Indicates which team performed the measurements. Missing values for this field: 0.00%. Possible values: B (52.22%), A (47.78%).
- **visit_date**: The date of a particular visit. Note that these were shifted per patient to preserve privacy. The relative time between visits of a patient are preserved. Format: YYYY-MM-DD. Missing values for this field: 0.00%. Range: 30/03/1984 - 02/02/2015.

#### Table: test

This table contains the metadata of the tests. A test in this context means a set of measurements for a specific limb. E.g. the test of the left arm will contain multiple measurements of said arm. This table is contained in the file test.csv.

**Key fields**:

- **patient_uid** - cfr. patient_uid in Table patient.
- **visit_uid** - cfr. visit_uid in Table visit.
- **test_uid**: A unique identifier for a test. It is only unique in conjunction with the patient, and visit uid. Range: 0–6.

**Other fields**:

- **anatomy**: The muscle on which the measurement is performed. Missing values for this field: 0.00%. Possible values: APB (50.71%), AH (49.29%).
- **side**: The side on which the measurement was performed, e.g., left or right arm. This value is generated based on the measurement protocol which says the tests must be performed on the right limb first, then the left limb. The accuracy of this field depends on how well the nurses stuck to this protocol. Missing values for this field: 0.00%. Possible values: R (50.45%), L (49.55%).

#### Table: measurement

This table contains the metadata of the measurements. These are the actual MEP measurements, i.e., the recordings of electrical activity at either the hands or feet after stimulation of the motor cortex using TMS. The actual time series can be found in separate text files, the filename of which is reflected in the *timeseries* field. This table is contained in the file measurement.csv.

**Key fields**:

- **patient_uid** - cfr. patient_uid in Table patient.
- **visit_uid** - cfr. visit_uid in Table visit.
- **test_uid** - cfr. test_uid in Table test.
- **measurement_uid**: A unique identifier for a measurement. It is only unique in conjunction with the patient, visit, and test uid. Range: 0–16.

**Fig. 6:**
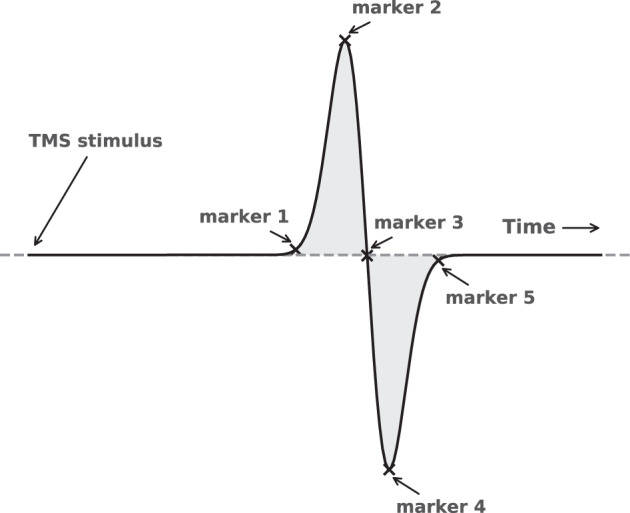
A simplified depiction of a MEP measurement for the illustration of the placement of the markers. Marker 1 is the point usually referred to as the *latency*, as illustrated in Fig. 2. The second marker indicates the first major peak in the signal, the third the first zero-line crossing, the fourth the first major low point, and finally the fifth marker marks the point at which the signal returns to the zero-line. The exact locations of these markers may be somewhat subjective in MEPs with strong fluctuations in the signal. Therefore, previous work has mainly focused on the first marker, i.e., the latency.

**Other fields**:

- **marker_N_amplitude(mv)**: Float field which indicates the amplitude of a certain marker, which were placed by nurses in the clinic. The placement of the markers are illustrated in Fig. 6. This should be used with the corresponding marker_N_latency(ms) field. Refer to Table 1 for some statistics of this key.

**Table 1:** Summary statistics of the available markers.

	AH	APB
**marker_1_amplitude(mv)**	−0.01 ± 0.06(7.75%)	−0.00 ± 0.06(2.71%)
**marker_1_latency(ms)**	43.15 ± 7.57(7.75%)	22.21 ± 4.92(2.71%)
**marker_2_amplitude(mv)**	0.78 ± 0.72(17.40%)	1.20 ± 1.09(4.80%)
**marker_2_latency(ms)**	53.08 ± 8.96(17.40%)	30.37 ± 6.63(4.80%)
**marker_3_amplitude(mv)**	0.04 ± 0.16(18.70%)	0.02 ± 0.17(5.13%)
**marker_3_latency(ms)**	55.41 ± 9.03(18.70%)	33.40 ± 7.28(5.13%)
**marker_4_amplitude(mv)**	−0.50 ± 0.42(18.77%)	−0.77 ± 0.66(5.14%)
**marker_4_latency(ms)**	59.22 ± 9.86(18.77%)	36.81 ± 9.32(5.14%)
**marker_5_amplitude(mv)**	−0.03 ± 0.10(18.89%)	−0.08 ± 0.15(5.36%)
**marker_5_latency(ms)**	76.35 ± 11.93(18.89%)	51.57 ± 10.59(5.36%)

Refer to Fig. 6 for the placement of the markers. The formatting is as follows: mean ± standard deviation (percentage missing). The statistics are shown separately for each of the possible anatomies. ms and mv indicate milliseconds and millivolts respectively.
- **marker_N_latency(ms)**: Float field which indicates the latency of a certain marker, which were placed by nurses in the clinic. The placement of the markers are illustrated in Fig. 6. This should be used with the corresponding marker_N_amplitude(ms) field. Refer to Table 1 for some statistics of this key.
- **notch_filter**: A boolean field indicating whether a 50 Hz notch filter was applied during the measurement. Missing values for this field: 0.00%. Possible values: False (81.42%), True (18.58%).
- **timeseries**: The full MEP timeseries. Missing values for this field: 0.00%. This field holds an integer identifier for the time series belonging to the measurement. The timeseries themselves are stored separately, with the filename corresponding to the identifier stored in this field. These timeseries measure the electrical signal received from the motor cortex in the muscle indicated in the **anatomy** field. The measurement always takes 100 milliseconds. Due to the different sample rates of the machines, the number of samples per timeseries differs slightly (1920 or 2000).
  This table contains the EDSS measurements. Not all patients are represented in this table. These measurements are frequently used as a target in machine learning tasks, both longitudinal and cross-sectional. This table is contained in the file edss.csv.
  **Key fields**:
  - **patient_uid** - cfr. patient_uid in Table patient.
  - **date** - See below.
  **Other fields**:
  - **date**: The date on which the EDSS measurement was performed. Format: YYYY-MM-DD. Missing values for this field: 0.00%. Range: 17/01/1982 - 04/05/2016.
  - **edss**: The result of the EDSS measurement. Missing values for this field: 0.00%. Average value: 3.28 ± 2.05

### Privacy

A number of steps were taken to ensure the privacy of the patients on whom these measurements were performed. Sensitive fields that were removed include the patient id used in the clinic and the names of the patients. The dates of the measurements were shifted by a random period of time, though in such a way that the relative time between measurements of a given patient is preserved. The same shift was applied to the dates of the clinical measurements. The patient’s date of birth was shifted in the same way the measurement dates were shifted to ensure the age at the time of measurement still matched. The resulting date of birth was then shifted by a random time interval with length within ±5 years.

The dataset was subsequently probed by an independent third party (P95) to assess the risk of re-identification. The steps described earlier were found to be sufficient for the dataset to be made available publicly.

## Technical Validation

The Rehabilitation and MS center is a treatment center which specializes in the day-to-day care of MS patients. Measurements are performed in a standardized fashion and are used in day-to-day clinical followup meaning they were reviewed by the neurologist treating the patient.

To check the validity of the final database, a number of sanity checks were automated.

Examples are the ages being in a valid rage, the sex of the patients being unique, and a reasonable number of visits per patient. If any systematic error was made during the extraction of the data, these checks are likely to fail. The time series were also compared to the reports that are generated by the software, which are the files the neurologists look at to assess the measurements. This was done for a random sample of the visits. These reports could be replicated exactly using our extracted database.

## Usage Notes

The measurements almost always include a measurement artifact at the start of the time series. This is caused by the electric field generated by the coil which is also picked up by the electrodes. This part can be safely discarded. In our work, we usually discard the first 70 points of any time series.

There are multiple measurements per test. Usually the measurement with the highest peak-to-peak amplitude is considered to be the most informative, which is what we used in our previous work. One should be careful to discard the previously mentioned measurement artifact when calculating the peak-to-peak amplitude, as this artifact usually has a larger amplitude than the relevant peaks in the time series.

Since the measurements were performed on two separate machines, a researcher using this dataset has two options. Either preprocess the time series to match the settings of the two machines (e.g. by applying the same filters to the machines). Alternatively, the researcher could opt to use the measurements in their current form and use the differences between the machines as regularization for their model, ensuring better generalization to multicenter studies. For the former approach, note that the two machines on which the measurements were made have slightly different sampling rates. Therefore, we suggest downsampling the time series from the machine with 2000 samples per time series to the 1920 samples of the other machine. The high-pass cutoff frequencies on both machines also differ. This could be rectified by applying a 100 Hz filter to the machine which originally has a 0.6 Hz filter applied to it.

There is a slight difference in some of the features of the time series recorded by either team (indicated in the field *team* in the *visit* table). For example, there is a difference in the average latencies of roughly 1 millisecond between the two teams. These differences were not significant enough to affect the interpretation of the neurologists. However, depending on which features are used to represent the measurements in a statistical model, this difference may be significant. A notable difference between measurements taken from different hospitals or different measurement devices is a common occurrence in a real-world setting, and an important hurdle for the translation of machine learning methods to the clinic^19^. Its occurrence in this dataset is therefore not atypical and is expected to also occur when MEP measurements from other MS centers are included. The development of machine learning techniques that are able to deal with such issues is of great importance.

In case no spontaneous response or MEP in rest position is obtainable, a light voluntary contraction of the muscle in question is asked in order to activate the motor cortex and increase the possibility of becoming a motor answer. This so-called facilitation method is usually very noisy due to baseline contraction of the muscle measured. Unfortunately, whether this method was used was not consistently indicated with each measurement. Facilitated measurements are characterized by a non-flat signal right from the start of the measurement. In our previous work^16,17^ we dropped any time series where the sum of absolute values in the interval between 5 milliseconds (to avoid the aforementioned measurement artifact) and 17 milliseconds (the earliest the signal can physiologically arrive for the hands) is above an empirically determined threshold. This threshold was chosen by visually inspecting the time series, and should therefore only be considered as a guideline. This calculation was done on the resampled time series (cfr. previous paragraph). Facilitated measurements are included in the dataset. There is code available in the dataset repository that removes the facilitated measurements using the described method (in sample_use_case.ipynb).

There are several possible use cases for this dataset. One can investigate the cross-sectional correlation of different MEP markers with the EDSS, which until now has mostly been done for the latency^20–24^.

Predicting disability progression after a certain number of years^25–28^ is of relevance to people with MS^29^. This has been done over a 2-year window on this dataset^17^.

Finding patient subtypes in an unsupervised manner could lead to interesting results^30^. One could investigate the degree to which this data is non-stationary, and if it is, how to correct for it^31^. This dataset contains sparsely measured visits (a few per year) of high-dimensional data, so it can be used to investigate the performance of machine learning algorithms that can handle this kind of data^32–36^. Such methods have already been applied to MS datasets^37,38^. More generally, methods developed to analyze electronic health records could be applied to this dataset^39^. For all these tasks, models could be optimized for interpretability^40^.

To illustrate how to use the dataset we have included a Jupyter notebook in Python in which a machine learning pipeline for the task of cross-sectional prediction of the EDSS is implemented. It highlights some of the choices that have to be made when selecting a suitable subset of the dataset to work with. We also provided an Microsoft Excel document for quick visualization of the data. Instructions on how to use these are included in the data repository.

## Data Availability

The code to generate the dataset is not available, as it deals with privacy sensitive source data. Alongside the dataset itself we also provide a Jupyter notebook in Python which implements a machine learning pipeline on the dataset for the task of cross-sectional prediction. This code illustrates how to handle the data. The notebook, some helper code and the data are available at https://gin.g-node.org/JanYperman/motor_evoked_potentials.
